# A scoping review of systems approaches for increasing physical activity in populations

**DOI:** 10.1186/s12961-022-00906-2

**Published:** 2022-09-29

**Authors:** Tracy Nau, Adrian Bauman, Ben J. Smith, William Bellew

**Affiliations:** 1 Prevention Research Collaboration, Charles Perkins Centre, School of Public Health, Faculty of Medicine and Health, The University of Sydney, Sydney, NSW Australia; 2 The Australian Prevention Partnership Centre, Sydney, NSW Australia

**Keywords:** Systems thinking, Systems change, Physical activity, Public health

## Abstract

**Introduction:**

The past decade has increasingly seen systems approaches as a featured theme in public health studies and policy documents. This trend is evident in the area of physical activity, which is a significant global health risk factor that is addressed in WHO’s Global Action Plan on Physical Activity. We undertook a comprehensive scoping review to characterize the application of systems approaches to physical activity, to develop a typology of the objectives, themes and methods of research papers that purported to apply systems thinking to this issue.

**Methods:**

We searched electronic databases (PubMed, Web of Science, Scopus and PsycINFO) for studies published during the period 2010–2021 that explicitly applied systems approaches or methods to investigate and/or address population physical activity. A framework using systems-based methodological approaches was adapted to classify physical activity studies according to their predominant approach, covering basic descriptive, complex analytical and advanced forms of practice. We selected case studies from retained studies to depict the current “state of the art”.

**Results:**

We included 155 articles in our narrative account. Literature reporting the application of systems approaches to physical activity is skewed towards basic methods and frameworks, with most attention devoted to conceptual framing and predictive modelling. There are few well-described examples of physical activity interventions which have been planned, implemented and evaluated using a systems perspective. There is some evidence of “retrofitted” complex system framing to describe programmes and interventions which were not designed as such.

**Discussion:**

We propose a classification of systems-based approaches to physical activity promotion together with an explanation of the strategies encompassed. The classification is designed to stimulate debate amongst policy-makers, practitioners and researchers to inform the further implementation and evaluation of systems approaches to physical activity.

**Conclusion:**

The use of systems approaches within the field of physical activity is at an early stage of development, with a preponderance of descriptive approaches and a dearth of more complex analyses. We need to see movement towards a more sophisticated research agenda spanning the development, implementation and evaluation of systems-level interventions.

**Supplementary Information:**

The online version contains supplementary material available at 10.1186/s12961-022-00906-2.

## Introduction

Interest in the use of systems approaches in public health has grown rapidly, with up to 90% of published examples emerging in the last 10 years [1, 2]. Recently, systems approaches have been recommended for increasing population levels of physical activity (PA), in recognition of the principle that the complex issue of physical inactivity cannot be addressed by simple, single solutions [3]. Historically, health promotion policy and programmes to address physical inactivity have come to rely on social ecological models to guide strategies at multiple levels, involving partners from diverse sectors of government and society [1, 4]. While systems approaches share some of the elements of social ecological models, they advance these concepts by their attention to the interrelationships within and across levels of influence, as well as the wide range of interacting outcomes from efforts to intervene in the system. These outcomes may be intended or unintended, positive or negative, amplified or diminished depending on how the system responds. Systems-based interventions to address PA may use established public health planning frameworks and strategies [4–6] but can also be informed by methodologies grounded in systems thinking or systems science; examples include system mapping, network analysis and system modelling [5, 6].

Despite the widely recognized potential for systems approaches to address complex public health issues like physical inactivity, reviews indicate that studies tend towards description rather than intervention and display a number of limitations [1, 2, 5, 7]. A range of barriers to the use of systems approaches in public health have been reported, including (i) lack of understanding about what they are, (ii) uncertainty about how to apply them in practice, (iii) perceptions that they are too difficult to apply or require new approaches and complex skills, (iv) scepticism about whether they add value sufficient to justify the required effort and resources, and (v) structural factors such as existing government processes and funding mechanisms which tend to reinforce the status quo [4, 5]. Without clear, practice-oriented guidance on how to implement and evaluate systems thinking and approaches, practitioners will continue to take the known options of interventions that address a limited array of risk factors believed to have a direct and linear influence on public health problems [8].

If the potential for systems approaches to PA is to be realized, there is a need for greater awareness, knowledge and skills among policy-makers and practitioners about how to apply these approaches as well as an understanding of their added value [5]. The peer-reviewed literature is a natural starting point for learning more about how systems approaches are currently understood and applied to PA. It is also a body of literature that can be systematically identified and accessed using current search practices, compared with grey literature which is more disparate. However, it is acknowledged that governmental and nongovernmental reports and other outputs may also offer important contributions towards systems thinking or practice [5]. Previous reviews have attended to public health in general terms, but an examination focused on PA has not been undertaken [2, 5, 6]. We attempt to address this gap by conducting a scoping review, which is widely accepted to be a suitable and rigorous approach for providing a descriptive overview of the state of research activity in a field, particularly in an area that is emerging, poorly known and dispersed across various methodologies and disciplines (as is the case for systems approaches for PA) [9–11]. Our objectives were firstly to describe and classify the major themes and strategic applications in the peer-reviewed literature that reported using systems approaches in PA, and secondly to provide case studies that illustrate systems approaches and the methods used.

## Methods

We conducted a scoping review, guided by methodological frameworks developed by Arksey and O’Malley [9] and Levac and colleagues [10], and reported according to the Preferred Reporting Items for Systematic Reviews and Meta-Analyses criteria for scoping reviews (PRISMA-ScR) [12] (Additional file 2).

### Search criteria

We searched electronic databases (PubMed, Web of Science, Scopus and PsycINFO) for studies published from 2010 to 2020 that reported using a systems approach or methods for enhancing population PA. Searches were run for all databases on 16 December 2020. Search terms were developed by consulting the search strategies in previous reviews of systems approaches for public health, and developing a search string that incorporated the explicit systems terms commonly used in previous reviews [2, 13, 14] ("systems approach*” OR “systems thinking" OR "whole-of-system*" OR "whole of system*" OR “whole system* approach*” OR "systems map*" OR “system* dynamic*” OR “systems science” OR “systems-based approach*” OR “complex systems”) combined with PA terms ("physical activity" OR "sport" OR “sports[MeSH Terms]” OR “walking” OR “cycling” OR “active travel” OR “active transport*” OR “exercise[MeSH Terms] OR “bicycl*” OR “biking” OR “active commut*” OR “public transport*”). We also considered the included studies of relevant reviews found using these search terms. Additional studies from 1 January 2021 until 31 December 2021 were identified from Web of Science notifications for newly published studies meeting the search criteria.

### Screening

The criteria for inclusion and exclusion are set out in Table 1. Studies could address PA as a component of a broader public health initiative such as obesity prevention, provided they addressed PA as a discrete component of the intervention or analysis.

**Table 1:** Inclusion and exclusion criteria

Inclusion criteria	Exclusion criteria
• English language • Peer-reviewed papers with available full text, including conceptual or theoretical papers and papers that synthesized existing literature • Explicitly identify as using systems approaches or methods, or describe an application of systems approaches to PA • Application of systems approaches to PA as a discrete component of the intervention or analysis	• Conference abstracts, dissertations, grey literature, book chapters • Application of systems approaches: - for sport injury, athletes or professional sport - to reduce road injury - to model pedestrian movements in crowded contexts - for physiological studies - for social justice purposes (e.g. sport for development and peace) - for transport (unless they addressed active transportation, i.e. public transport, walking and cycling)

We adopted a conservative approach, and papers were retained for full-text screening if it was unclear from the title and abstract whether inclusion criteria were met. A subset of 200 papers were screened independently by two authors (TN and AB), with an inter-rater agreement of 94.5%. Conflicts were resolved by discussion and agreement between TN and AB. A further 83 papers were screened jointly to achieve consensus on decision criteria, and then each paper was screened by one reviewer. The full-text versions of the papers were screened by one reviewer (TN), with any uncertainties resolved by discussion with other authors (WB and AB). Papers that related to the same study (e.g. the WHO STOPS trial [15–17]) or related to the same overarching initiative (e.g. the Healthy Kids, Healthy Communities [HKHC] projects funded by the Robert Wood Johnson Foundation) [18–22] were included and classified separately according to the stage of systems approach and method used.

### Data charting

For each paper, one reviewer (TN) documented the author/year, title and a description of how the study applied a systems approach for PA.

#### Classification of articles

We adapted an existing framework of systems methods for public health evaluations [6] to classify PA studies according to the predominant methodological approach they used (system mapping, network analysis, system modelling, system framing, protocol development, generic methods, methods development and literature synthesis) and the strategic intent of their systems approach (theorizing, prediction [simulation], intervention development, process evaluation, impact evaluation).

Additional categories were created to capture articles that did not correspond to the existing categories in this framework (i.e. intervention development, protocol development, methods development, literature synthesis) (Table 2). Although we have proposed “stages” of a systems approach, this was mainly to provide a classification framework for the different applications of systems thinking and methods to PA, rather than to denote a progression or hierarchy of approaches. We classified studies as “unclear systems approaches” if, based on the information provided, we could not determine the systems perspective or method being used.

**Table 2:** Typology of systems methods used in physical activity research across stages of systems approaches

Stage of systems approach	Aim	System mapping^a^	Network analysis^b^	System modelling^c^	System framing^d^	Protocol development^e^	Generic methods^f^	Methods development^g^	Literature synthesis^h^
Theorizing	Identify and compare stakeholder understanding of a complex system	X	X		X	X			
	Identify and compare stakeholder understanding of how a planned/hypothesized intervention might interact within a complex system	X			X				
	Explore the role, application or implications of using systems approaches or methods in a particular context			X	X			X	X
Prediction (simulation)	Hypothesize and simulate how an intervention may impact on and interact with a complex system	X	X	X			X		
	Hypothesize and simulate how agents within a complex system react and interact in response to an intervention			X				X	
Intervention development (formative)	Design interventions for real-world implementation within a complex system	X			X	X			
Process evaluation	Understand how an implemented intervention interacts with and influences a complex system in the real world	X			X	X	X		
Impact evaluation	Quantify the impacts or outcomes of an implemented intervention on key system parameters in the real world						X		X

The “X” symbols in this table denote categories where papers were found in this review

^a^System mapping: studies that theorize and illustrate a system's boundaries and interrelated parts

^b^Network analysis: studies that focus on relationships between individuals or organizations relevant to a system

^c^System modelling: computational models that simulate changes within a complex system over time

^d^System framing: approaches that have emerged from the systems thinking tradition or from attempts to apply systems theories and concepts to other public health issues

^e^Protocol development: studies that describe the design or methods that will be used for a particular stage of a systems approach

^f^Generic methods: studies that primarily apply non-systems methods to a particular stage of a systems approach

^g^Methods development: studies that primarily describe the development or refinement of methods or tools to support a systems approach

^h^Literature synthesis: uses a systematic or narrative approach to review published literature on systems approaches or methods

Three authors (AB, WB, TN) classified each paper according to the adapted framework based on the dominant category that it corresponded to for the stage of systems approach, and the dominant category for the systems methods used. In the few instances where papers could be classified under multiple categories, the authors conferred and reached consensus on the appropriate classification. Following classification, a numerical summary analysis was conducted along with a narrative overview of the types of studies found for each stage of systems approach and the methodological approaches used within each stage.

Fisher’s exact test was used to examine the significance of any differences in the distribution of publications for each category of systems approach and method used, across two time periods, 2010–2015 and 2016–2021. We purposively selected case studies from the included studies that clearly described their use of systems methods for a particular stage of systems approach for PA.

## Results

**Fig. 1:**
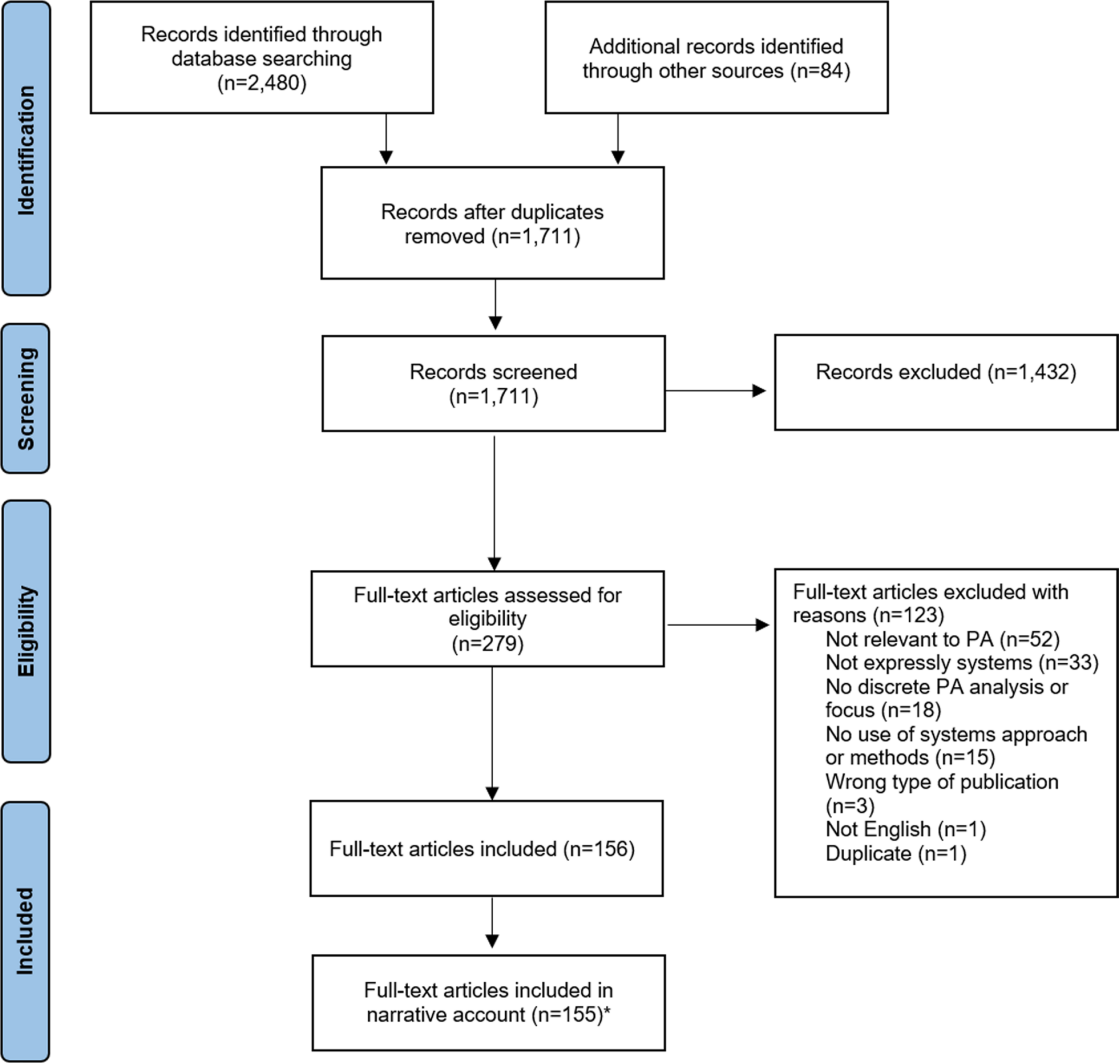
Flow diagram for inclusion of studies. *The number of full-text articles included in the narrative account was 155 rather than 156, as one of the articles was an authors’ reply to a response provided to an earlier publication

Our review included 155 publications for the following narrative account from 2480 identified through the literature search. Figure 1 presents a flow diagram of publication identification, screening, eligibility assessment and inclusion.

In Additional file 1: Tables S1–S5 show the information extracted from each study, organized by stage of systems approach, and Additional file 1: Table S6 identifies those studies that were classified as unclear systems approaches.

Table 3 summarizes the number of publications found for each stage of systems approach and method. The predominant category was Prediction publications (*n* = 61), which mainly used system modelling approaches, followed by Theorizing publications (*n* = 61), which mainly used system framing approaches (*n* = 22). There were 11 Intervention development publications, mainly protocol papers (*n* = 5), and 11 Process evaluation publications, mainly using system framing (*n* = 7). The least common category was Impact evaluation publications (*n* = 8), and these mainly used generic methods (*n* = 7).

**Table 3:** Distribution of the number of publications across stages of systems approach and type of methodological approach

	Methodological approach								
Stage of systems approach	System mapping	Network analysis	System modelling	System framing	Protocol development	Generic methods	Methods development	Literature synthesis	Totals (stages of systems approach) [*N* = 155]
Theorizing	16	5	1	22	1	0	5	11	61
Prediction	2	1	54	0	0	1	3	0	61
Intervention development	2	0	0	4	5	0	0	0	11
Process evaluation	1	0	0	7	1	2	0	0	11
Impact evaluation	0	0	0	0	0	7	0	1	8
Unclear systems approach	NA	NA	NA	NA	NA	NA	NA	NA	3
Totals (methodological approaches) [*n* = 152^a^]	21	6	55	33	7	10	8	12	

^a^The total is 152, 3 less than the total number of publications (*N* = 155), as unclear approaches were not classified by method

**Fig. 2:**
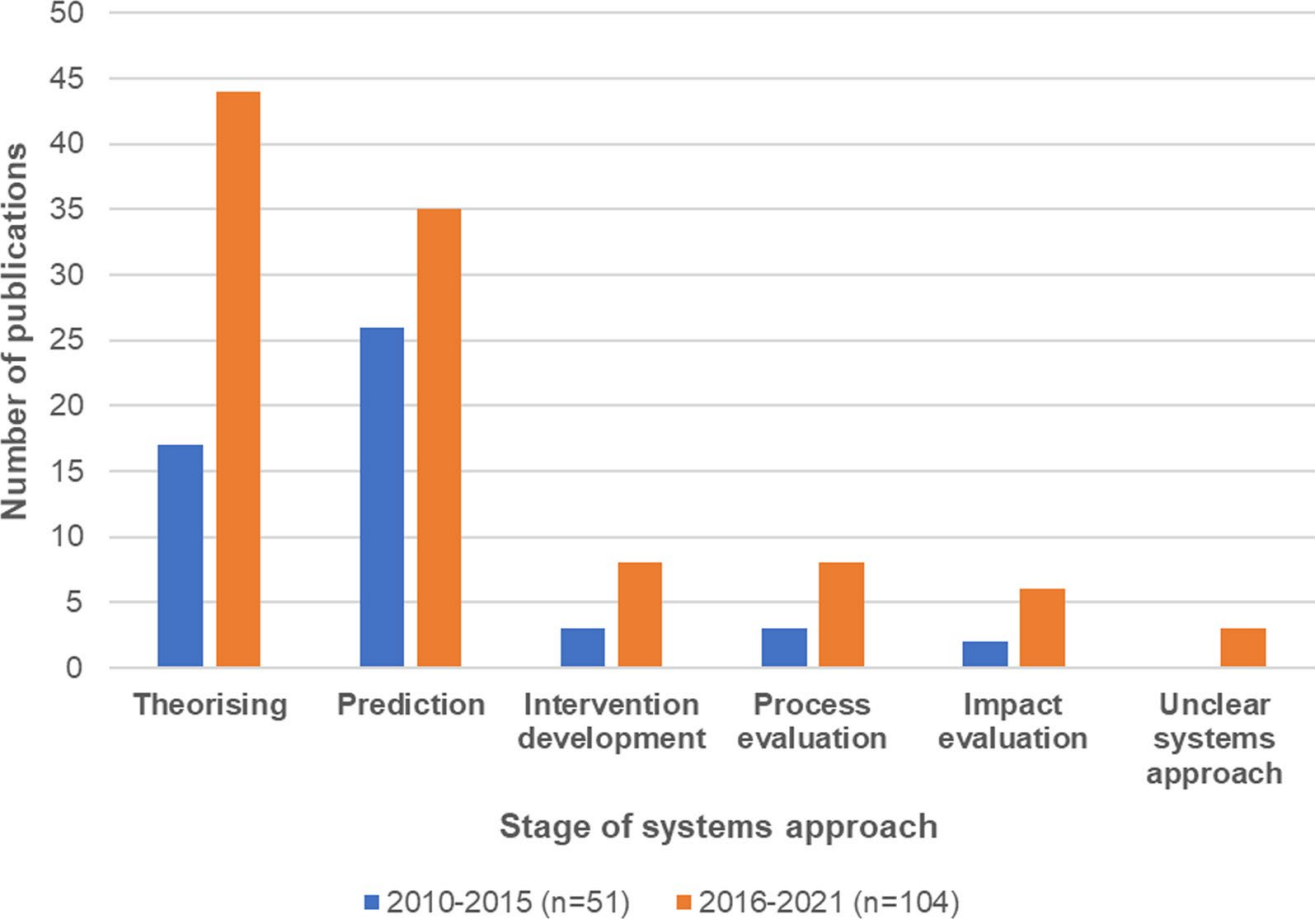
Distribution across different stages of systems approach by number of publications from 2010–2015 to 2016–2021

**Fig. 3:**
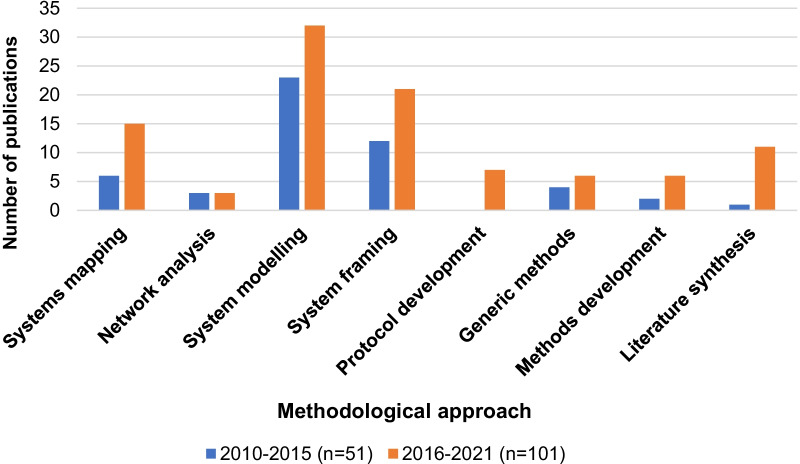
Distribution across different methodological approaches by number of publications from 2010–2015 to 2016–2021

Figures 2 and 3 compare the distribution of publications across different stages of systems approaches and method used from 2010–2015 to 2016–2021. They show an increase in the number of publications across all stages of systems approaches, with Theorizing overtaking Prediction as the most prevalent category in the most recent 5 years. They also show an increase in the use of each methodological approach (except for network analysis), although system modelling was still the most frequently used method. Despite the increase in some categories, the proportion of publications in each category was reasonably stable and not significantly different across the periods.

Further details about the articles found for each stage of systems approach and methods applied are provided below.

### Theorizing

Sixty-one articles were classified under the Theorizing stage of a systems approach, primarily using system framing (*n* = 22) and system mapping approaches (*n* = 16).

The aims of theorizing include identifying and comparing stakeholder understanding of a complex system [6] (Table 2), commonly to identify the drivers of PA and inactivity and relationships between them. System mapping was a widely used method for doing this. PA was the focus of the system map in some studies [23–29], while in others it was one of multiple components (e.g. along with healthy eating [20, 22, 30], or other risk factors for obesity [31] and diseases [32]). In some cases, the system map was developed entirely through group-based approaches [20, 23, 32]; in others, a preliminary map was provided for others to modify and build upon [26, 29]. It was commonly reported that the system map was used to identify opportunities to intervene and to help develop policies and interventions [20, 23, 26, 27, 30]. System framing was also used to support formative work to better understand existing systems, as a way of informing future systems approaches and interventions [33–35].

Social network analysis was used in five studies to identify and compare stakeholder understanding of their relationships with other individuals or organizations in a particular system. Understanding the types and functions of networks, and the role that key actors play, is considered useful for understanding where and how to intervene in a system [36, 37]. It has the potential to help identify influential leaders who may be champions for the intervention; leverage existing network capacity for information, policy or change diffusion [38]; and identify where existing networks may need to be formed or strengthened to improve the implementation of interventions [38, 39].

There were a few examples of developing new methods to support understanding about a particular system relevant to PA. For example, a new coding system was developed by Hoehner and colleagues [19] to aggregate and aid analysis of a large number of behaviour-over-time graphs that had been generated during group-based modelling sessions held in diverse communities involved in the HKHC initiative. Other studies that were categorized as Methods development aimed to guide the future design, monitoring and evaluation of systems interventions [40, 41].

Another aim of theorizing is to explore the role, application or implications of using systems approaches or particular methods in a particular context (Table 2). These studies were mainly classified as using system framing or literature syntheses. In relation to system framing, systems-based theories or frameworks (such as the intervention-level framework) were drawn on or developed to analyse existing strategies and policy and identify areas where they could be strengthened for obesity prevention [42, 43]; provide support for arguments about the need for a systems approach for PA [44] and diseases such as type 2 diabetes [45]; show how urban planning for walking and cycling contributes to the United Nations’ Sustainable Development Goals [46]; and explain how active lifestyles could be coproduced using a whole system approach [47]. Most of the included literature syntheses for the Theorizing category reviewed applications of system dynamics modelling (SDM) or agent-based modelling (ABM) in public health, chronic disease and obesity [48–52]. Two studies were focused on PA in terms of assessing the use of simulation modelling to inform decision-making about built environment influences on PA [53], and examining the use of systems-based simulation studies to evaluate the health-related consequences of active transport [54].

Case study 1. System mapping “*Using system mapping to help plan and implement city-wide action to promote physical activity*” [29]

**Aim:** To investigate whether system mapping could be a useful tool to help improve the planning and implementation of a city-wide PA promotion programme in Derby, United Kingdom, by promoting the use of systems thinking.

**Methods:** The authors initially produced draft conceptual maps of the major modifiable drivers of PA in the city, based on existing literature on the determinants and correlates of PA, which were refined in a series of stakeholder meetings. The maps were used to explore ways in which the existing programme adopted a systems approach, existing data sources that could be used to measure the impact of the programme, and actions that could be undertaken to improve the delivery of a systems approach. Semi-structured interviews were conducted with stakeholders to assess their views on the contribution of the mapping approach.

**Findings:** The stakeholders described the mapping as valuable, particularly for identifying the limitations of the original approach taken in the city. The authors reported that even a simple application of systems thinking can be a useful tool for disaggregating key factors in a system, helping to identify areas that need greater attention and supporting effective action.

### Prediction (simulation)

Sixty-one articles were classified under the Prediction stage of a systems approach, mostly system modelling studies (*n* = 54).

The aims of prediction include hypothesizing and simulating how an intervention might impact on and interact in a complex system [6] (Table 2), which was typically achieved in the included studies using SDM. Many SDM studies were conducted in relation to active transport (*n* = 10) [55–64]. Several tested the effects of different policies or interventions on outcomes such as cycling, walking, public transport and active transport to school [57, 59, 61, 64], in some cases together with other outcomes such as injury, fuel costs, air pollution and carbon emissions [57, 59]. In other studies, the SDM focused on the effects of different policies and interventions on individual transport mode choice [62], with the main consideration being reducing carbon emissions, improving mobility efficiency and sustainability [58, 62, 63] or reducing motor vehicle deaths and injury [60].

A number of studies used SDM to simulate the effect of PA behaviours or policy interventions on obesity outcomes (*n* = 7) [65–71]. Several (*n* = 3) used the Prevention Impacts Simulation Model (PRISM) (an SDM) to project the reduction in deaths and costs from PA promotion and other obesity prevention efforts [72–74].

Another aim of prediction is to hypothesize and simulate how agents within a complex system react and interact in response to an intervention [6], using ABM (*n* = 26) [75–100]. Many of these studies were focused on active transport and/or built environment interventions and simulating their potential interaction with agent characteristics (e.g. their walking ability, attitudes to different transport modes, vehicle ownership, social networks) to model their impact on outcomes such as walking, cycling, public transport and mode share [76, 83–85, 90, 92–98, 100].

A selection of studies used ABM to explore the potential impact of interventions on PA in children [75, 81, 95, 99]. These included interventions relating to a combination of outdoor play, school physical education and active travel [75]; using dynamic furniture in the school environment [81]; and the operation of after-school programmes [99]. Some studies had an equity focus, for example to explore the impact of intervention scenarios on income inequalities in sports participation [80]; and to simulate the effects of PA infrastructure on reducing racial disparities in BMI [88]. There were limited examples of methods development (*n* = 3) [101–103]. These included developing a participatory approach to codesign an ABM about PA with adolescent youth [101] and a new methodology for representing walking behaviours and benchmarking agent movement between models and against real-world data [103].

Case study 2. System modelling “*Using simple agent-based modeling to inform and enhance neighborhood walkability*” [78]

**Aim:** To develop an open-source, simple agent-based walkable catchment tool that can be used by researchers, urban designers, planners and policy-makers to test scenarios for improving neighbourhood walkable catchments prior to developing new or retrofitting older areas.

**Methods:** The initial development of the tool was informed by the health and place-based literature, earlier research to apply and test the walkability index with various health outcomes, and a prototype tool developed for pedestrian catchment analysis. This was supplemented by information provided by a stakeholder working group comprising representatives from Australian federal, state and local government agencies from the transport, planning and health sectors.

**Findings:** The resulting model allows stakeholders to assess and optimize the walkability of neighbourhood catchments around actual or potential nodes of interest (e.g. schools, public transport stops). A range of metrics can be used to compare different scenarios that are modelled, including mean number of streets crossed, different walking speeds and wait time at intersections. The tool has the potential to be influential as a planning and public health advocacy tool for the development of more walkable and accessible neighbourhoods, and around key destinations of interest.

### Intervention development

This category concerned the development of interventions for real-world implementation within a complex system. Our review found 11 articles in this category [15, 18, 21, 104–111], many of which were protocols (*n* = 5) [15, 108–111], although one of these protocols was subsequently implemented [15] and two others were written part-way through programme implementation [109, 110]. Most of the protocols focused on childhood obesity prevention [15, 108–110] and were set in towns or cities in Australia [15, 108] or England [109, 111]. Several referred to participatory system mapping or group model-building as one of the methods to inform intervention development [15, 18, 21, 108, 110, 111].

Four studies were categorized as using system framing to develop interventions for PA [104–107]. One of these studies used a systems lens to provide insights into how the Government of South Australia used a Health in All Policies approach to develop high-level policy commitments for PA (and other factors) [106]. Another study used a mixed-methods design to evaluate and refine a community-based, systems approach to childhood obesity prevention, called Live 5–2-1–0 [104].

Case study 3. System framing “*Controlled before-after intervention study of suburb-wide street changes to increase walking and cycling: Te Ara Mua-Future Streets study design*” [105]

**Aim:** To develop a best-practice walking and cycling infrastructure intervention in a suburb with a high proportion of low-income residents and a high proportion of residents experiencing inequities associated with ethnicity (particularly Māori—New Zealand’s indigenous peoples—and Pacific peoples).

**Methods:** Used best-practice community codesign for the infrastructure intervention, and triangulated community knowledge with high-quality evidence, to develop interventions that were contextually and culturally appropriate. An iterative process of engagement and revision was used to develop the final infrastructure intervention designs.

**Findings:** Interventions included a range of infrastructure changes to reallocate road space from vehicles to pedestrians and cyclists; improve street crossing safety and convenience; improve the safety of routes through parks; and landscaping to reflect indigenous culture and history.

### Process evaluation

Process evaluation involves understanding how an implemented intervention interacts with and influences the PA system in the real world [6] (Table 2). Most of the 11 studies in this category were classified as using system framing (*n* = 7) [8, 16, 112–116], with the remaining studies using system mapping (*n* = 1) [117] generic methods (*n* = 2) [118, 119], or proposing methods for process evaluation in a protocol (*n* = 1) [120].

Several studies used system framing to provide a unifying approach to analysing data from multiple sources, so that the factors and processes contributing to the implementation of an intervention could be better understood [112–115]. One study applied Foster-Fishman’s theoretical framework to describe actions taken to drive systems change in two communities to improve children’s health [16]. The components of the framework used were focused on system norms, operations and regulations. Another study used a multilevel perspective conceptual framework as a guide for mixed-methods analysis to elucidate the process of embedding a public bike-sharing scheme into the physical, social and institutional fabric of a city [115].

An example of using generic methods for process evaluation was the use of the validated Community Readiness Tool to assess whether a whole-of-community, systems-level obesity prevention initiative (known as YCHANGe) in a rural community in Australia improved the level of community readiness to change over time [119].

Case study 4. Protocol development “*A whole system approach to increasing children's physical activity in a multi-ethnic UK city: a process evaluation protocol*” [120]

**Aim:** To describe the protocol for a process evaluation of the JU:MP programme, a whole-systems approach to increasing PA in children and young people in North Bradford, United Kingdom. The aims of the evaluation were to understand the programme implementation and mechanisms through which JU:MP influences behaviour change across the neighbourhood, and wider policy and strategy systems.

**Methods:** The process evaluation is underpinned by realist principles which emphasize the role of context. A mixed-methods approach is proposed, including semi-structured interviews, observation, documentary analysis, surveys and participatory evaluation methods including reflections and ripple effect mapping. There are three distinct but interrelated packages of work, at the strategic, neighbourhood and end-user level.

**Findings:** The paper advances knowledge regarding the development of process evaluations for evaluating systems interventions. The evaluation will also facilitate dynamic system change by providing feedback and contributing to iterative programme development.

### Impact evaluation

This category was defined as quantifying the impact or outcomes of an implemented intervention on key system parameters in the real world [6] (thus excluding any modelling studies that forecasted impact in hypothetical scenarios) (Table 2). There were few studies that conducted impact evaluation of a systems approach (*n* = 8) [17, 121–127], and most used generic methods (*n* = 7) [17, 122–127]. The impact evaluations mostly involved analysis of changes in PA behaviours resulting from systems approaches for obesity prevention [17, 123, 124, 126], although one study examined the impact on the health promotion activity and orientation of sports clubs [125].

Case study 5. Generic methods “*Four-Year Behavioral, Health-Related Quality of Life, and BMI Outcomes from a Cluster Randomized Whole of Systems Trial of Prevention Strategies for Childhood Obesity*” [17]

**Aim:** To test the effectiveness of the Whole of Systems Trial of Prevention Strategies for Childhood Obesity (WHO STOPS Childhood Obesity), a cluster-randomized trial of 10 communities randomly allocated to start intervention in 2015 or in 2019 (after 4 years) in South-West Victoria, Australia.

**Methods:** Data were collected from participating primary schools in 2015, 2017 and 2019, including self-reported PA. These data were used to determine adherence to Australia’s 24-hour movement guidelines. Data were also collected about the mode of transport that participants usually took to get to and from school, so that they could be classified as using active transport or not.

**Findings:** The number of children meeting PA guidelines increased by 8.2% between 2015 and 2019 within intervention communities but not in control communities. There were no significant changes in active transport.

### Unclear systems approaches

There were three examples of unclear systems approaches [128–130], primarily because the study, in our view, did not clearly describe how they used systems thinking or systems approaches in the development or delivery or their intervention. For example, the Moving Healthcare Professionals programme to embed prevention and PA into clinical practice did not clearly describe how systems thinking or systems approaches were used in the education delivery strategies [128]. The First 1000 Days programme for pregnant women to prevent obesity and related risk factors was similarly classified because it was focused primarily on individual behaviour change strategies and did not describe how systems thinking or approaches were applied [130].

## Discussion

The role of systems thinking and accompanying tools such as system mapping in helping to frame responses to complex public health challenges has grown in the past decade, with particular prominence in obesity prevention [27]. In 2018, the release of WHO’s Global Action Plan on Physical Activity (GAPPA) signalled that an important change in thinking had occurred, stipulating that “effective national action to reverse current trends and reduce disparities in PA requires a ‘systems-based’ approach” [3]. Our scoping review shows how systems approaches have been applied to PA prior to and since this call to action.

All the retained publications in this review reported incorporating systems approaches. However, it appears that few engaged robustly with systems concepts, and in particular with the unique properties of systems approaches that distinguish them from the social ecological models that have long guided PA research and interventions. Systems approaches are characterized by recognition of feedback and adaptation, dynamic interacting elements, nonlinearity, self-organization and emergence [4, 5, 7, 121]. The body of PA literature that sought to apply systems approaches emphasized theorizing—understanding the system (mapping) and prediction (modelling). There was an apparent lack of a cross-sectoral perspective (other than in the studies using community-wide approaches), a strong focus on built environment determinants, and little or no attention to the analysis of the subsystems of policy-making and some of the sectors that potentially have a significant role to play in efforts to promote PA (e.g. primary care, sport) [131]. It is hard not to conclude that some authors are “dedicated followers of fashion”, since they appear to have “retrofitted” complex systems methodological framing to describe programmes and interventions which were not necessarily designed as such. This is particularly the case for those papers categorized as unclear systems approaches.

The extensive use of system mapping to theorize PA determinants and potential intervention points in our review demonstrates how this is being adopted and perceived as valuable, as a method for generating shared understanding and priorities for PA promotion among diverse stakeholders who may otherwise be dispersed and disconnected in the system. The more limited application of social network analysis in theorizing studies suggests it may be underutilized as a tool for exploring how organizational interactions, through information sharing, coordination and cooperation, could be improved through analysis of the programme delivery environment using a systems lens. There is also potentially an opportunity for making greater use of social network analysis to help develop and target strategies to strengthen governance arrangements, a key systems-level intervention.

The limited application of systems methods for evaluation of PA interventions is consistent with the findings of the review led by McGill [6] concerning public health policy and programmes more broadly. That review did, however, offer examples of the potential applications of systems methods at different stages of evaluation. For instance, system framing can be used in process evaluation to gain insights from stakeholders about how an intervention interacted with different elements of the wider system [6]. A complex systems perspective can also be applied to conducting process evaluation with qualitative methods [132]. Network analysis can be used in impact evaluation as a tool for evaluating the impacts of interventions on social relationships in schools, workplaces and other settings [6]. Other methods such as participatory action research and qualitative comparative analysis may also be applied to evaluation from a system lens, but are yet to be well described. The use of systems approaches for evaluation appears to be an area for future methodological development [6].

Numerous papers in this review focused on modelling and simulations; however, it was usually not reported whether or how the resulting information informed decision-making and novel policy actions, although this may not always be clear given the complexities of policy development to address PA [35, 133]. The few applications of systems methods in PA intervention delivery and evaluation also showed that there remains a need to demonstrate whether this approach can lead to better intervention selection, engagement of strategic intersectoral partners in implementation, and generation of new forms of knowledge to improve policy and programme impacts to address this health priority. Other reviews of the use of systems approaches and methods in public health have similarly found limited practical applications and translation of these into impact and change [2, 13, 121, 134]. When WHO included “active systems” as one of four main strategies in GAPPA, this was further described as taking action on “governance, leadership, multisectoral partnerships, workforce capabilities, advocacy, information systems and financing mechanisms across all relevant sectors” [3]. These are whole-of-system level interventions, to which legislation and regulation [135] and system surveillance [136, 137] could arguably be added.

A strength of this review was its use of inclusive search methods and a peer-reviewed framework to identify and classify applications of systems approaches and methods for enhancing population PA, which has not been examined in previous reviews of systems approaches. However, it is possible that our typology does not reflect all the ways in which systems approaches may be adopted and developed over time. Our review was also limited to the peer-reviewed articles. It is likely that relevant examples of systems approaches or use of systems methods for population PA exist outside the formal academic literature. For example, the emergence of guidance on systems thinking, such as the suite of documents developed for civil servants by the United Kingdom Government Office for Science [138] and Getting Australia Active III which was developed primarily for Australian policy-makers [139], may lead to increased adoption of systems approaches in government settings. In future, a more comprehensive synthesis would need to rely on collaborative networks and additional search and extraction methods to enable such evidence to be synthesized [5]. More generally, while a scoping review was appropriate to meet the objectives of this study, it is subject to limitations that are typical of this approach; for example, we did not appraise the methodological quality of the studies included in this review [9].

### Conclusion and implications for research and practice

The use of system approaches to increase PA in populations is at a relatively early stage of development, with a preponderance of descriptive approaches and a dearth of more advanced forms of practice and analysis. The field needs to move towards more sophisticated research agenda encompassing the development, implementation and evaluation of system-informed approaches, and demonstrating their effectiveness and added value. This will require greater application of mixed-methods evaluation approaches. The design and evaluation of systems approaches for PA should also extend beyond setting-level interventions and address systems-level enablers that arguably include governance and leadership, legislation and regulation, multisectoral partnerships, workforce capabilities, advocacy, information systems, system surveillance and financing mechanisms. Discussion, formulation and evaluation of these strategic interventions remain under-investigated and should be a priority for future practice and research.

## Supplementary Information

**Additional file 1: Table S1.** Summary of included studies classified as Theorizing. **Table S2.** Summary of included studies classified as Prediction. **Table S3.** Summary of included studies classified as Intervention development. **Table S4.** Summary of included studies classified as Process evaluation. **Table S5.** Summary of included studies classified as Impact evaluation. **Table S6.** Summary of included studied classified as Unclear systems approaches.

**Additional file 2.** Preferred Reporting Items for Systematic Reviews and Meta-Analyses extension for Scoping Reviews (PRISMA-ScR) Checklist – completed for the scoping review of systems approaches for increasing physical activity in populations.

## Data Availability

The authors can confirm that all relevant data are included in the article and/or its additional information files.

## Abbreviations

ABM: Agent-based modelling
GAPPA: Global Action Plan on Physical Activity
HKHC: Healthy Kids, Healthy Communities
PA: Physical activity
SDM: System dynamics modelling
