# Supplementary material for: A scoping review of systems approaches for increasing physical activity in populations
Source: Health Res Policy Syst. 2022 Sep 29;20:104. doi: 10.1186/s12961-022-00906-2 (PMC9524093; doi:10.1186/s12961-022-00906-2)
Supplement: Supplementary file 1 — Additional file 1: Table S1. Summary of included studies classified as Theorizing. Table S2. Summary of included studies classified as Prediction. Table S3. Summary of included studies classified as Intervention development. Table S4. Summary of included studies classified as Process evaluation. Table S5. Summary of included studies classified as Impact evaluation. Table S6. Summary of included studied classified as Unclear systems approaches. [file 12961_2022_906_MOESM1_ESM.docx]

#### ADDITIONAL FILE 1

#### Supplementary Table 1. Summary of included studies classified as ‘Theorising’.

| Author(s) and year | Title | Description of the application of a systems approach for PA | Methodological approach |
| --- | --- | --- | --- |
| THEORISING |  |  |  |
| Allender et al 2015 | A community based systems diagram of obesity causes | Conducted GMB sessions to create a CLD representing community perceptions of determinants and causes of childhood obesity in a rural Australian community. The CLD was broken down into four domains: social influences, fast food and junk food, sport participation, and general PA. | System mapping |
| Bellew et al 2020 | Whole of systems approaches to physical activity policy and practice in Australia: The ASAPa project overview and initial systems map | Developed a conceptual whole of systems map for PA to guide progress beyond theoretical to application in the real world. | System mapping |
| Carlson et al 2012 | Complexity in built environment, health, and destination walking: A neighborhood-scale analysis | Proposed a conceptual model describing the complex feedback relationships between destination walking and public health, with the built environment expected to increase/decrease the strength of the feedback loop. | System mapping |
| Cavill et al 2020 | Using system mapping to help plan and implement city-wide action to promote physical activity | Worked with stakeholders in a city-wide PA promotion program in Derby, UK to investigate whether a conceptual map of the local PA system could be a useful tool to help improve the planning and implementation of the program. | System mapping |
| Chastin et al 2016 | The SOS-framework (Systems of Sedentary behaviours): An international transdisciplinary consensus framework for the study of determinants, research priorities and policy on sedentary behaviour across the life course: A DEDIPAC-study | Used concept mapping to develop a transdisciplinary dynamic framework, grounded in a system-based approach, to guide research on determinants of sedentary behaviour across the life span and intervention and policy planning and evaluation. | System mapping |
| Guariguata et al 2021 | Systems science for developing policy to improve physical activity, the Caribbean | Held GMB workshops with key stakeholders in the Caribbean region to develop a CLD to describe the system driving the increasing physical inactivity in the region and envision the most effective ways of intervening in that system to encourage and promote PA. Used the CLD to inform how the WHO Global Action Plan on Physical Activity might be adapted to a local context. | System mapping |
| Holdsworth et al 2017 | Developing a systems-based framework of the factors influencing dietary and physical activity behaviours in ethnic minority populations living in Europe – a DEDIPAC study | Used a concept mapping approach guided by systems thinking to develop a systems-based framework of factors influencing dietary and PA behaviours in ethnic minority populations in Europe to inform research prioritisation and intervention development. | System mapping |
| Keane et al 2015 | Healthy Kids, Healthy Cuba: Findings from a group model building process in the rural Southwest | Conducted a GMB session with 12 Healthy Kids, Healthy Cuba (HKHCuba) partners to develop BOTGs and a CLD to elicit the policy, systems or environmental influences on healthy eating and PA within the HKHCuba system and determine causal relationships between them. | System mapping |
| Ma et al 2020 | Using Collective Intelligence to identify barriers to implementing and sustaining effective Fundamental Movement Skill interventions: A rationale and application example | Pilot study that trialled Collective Intelligence — an applied systems science approach — to understand the barriers to the adoption, implementation and institutionalisation of effective FMS interventions for children and adolescents. | System mapping |
| Ma et al 2021 | Enhancing the implementation and sustainability of fundamental movement skill interventions in the UK and Ireland: lessons from collective intelligence engagement with stakeholders | Used Collective Intelligence with stakeholder groups to understand barriers to the implementation of FMS interventions, interdependencies between these barriers, and options to overcome the system of barriers identified. | System mapping |
| McGlashan et al 2018 | Comparing complex perspectives on obesity drivers: action-driven communities and evidence-oriented experts | Presented a quantitative comparison between the Foresight (expert-developed) systems map and a community-developed map of obesity drivers. | System mapping |
| Muñoz-Prieto et al 2018 | Application of the NEOH framework for self-evaluation of One Health elements of a case-study on obesity in European dogs and dog-Owners | Performed an internal evaluation among the actors of the One Health obesity and dog ownership initiative, using an evaluation framework developed by the Network for Evaluation of One Health. The framework covered the following elements: (1) the context – comprising the system, its boundaries and the OH initiative as a subsystem; (2) assessment of expected outcomes based on the theory of change; (3) process assessment of the operations and supporting infrastructures. | System mapping |
| Murphy et al 2021 | “Getting Ireland Active”—Application of a systems approach to increase physical activity in Ireland using the GAPPA framework | Reported on a process that facilitated a systems approach for identifying current good practice and gaps for promoting PA in Ireland. Elements of participatory action research were enabled through 3 stages: (1) aligning examples of actions from Irish policy documents to the GAPPA, (2) workshop with stakeholders across multiple sectors, and (3) review of outputs. | System mapping |
| Rutter et al 2019, 2020 | Systems approaches to global and national physical activity plans (and authors’ reply – Systems approaches to support action on physical activity) | Developed a conceptual systems map to illustrate the main drivers of PA and inactivity and the relations between them. | System mapping |
| Thomas and Reilly 2015 | Group model building: A framework for organizing healthy community program and policy initiatives in Columbia, Missouri | Conducted a GMB session with members of the multidisciplinary Healthy Community Partnership of Colombia, Missouri. Participants produced BOTGs illustrating the evolution of community variables related to child obesity, which were used to develop a CLD as a visual representation of the interacting systems. | System mapping |
| Uleman et al 2020 | Mapping the multicausality of Alzheimer’s disease through group model building | Applied systems thinking to map out known causal mechanisms and risk factors and develop a CLD for Alzheimer’s disease (PA being one of the variables). | System mapping |
| Jancey et al 2021 | Understanding prevention networks in a Local Government Area: Insights from a social network analysis Among Western Australian nutrition, physical activity, and obesity prevention programs | Used SNA to understand organisational network relationships in a Western Australian local government area, to inform discussions, policy, and practice to improve PA, nutrition and overweight/obesity prevention activities. | Network analysis |
| Marks et al 2013 | Using social network analysis to identify key child care center staff for obesity prevention interventions: a pilot study | Pilot study to determine the feasibility and relevance of SNA for child obesity prevention among staff within a long day care setting, by identifying childcare staff networks and key players that potentially influence long day care dietary and PA planning. | Network analysis |
| Marks et al 2018 | Networks for prevention in 19 communities at the start of a large-scale community-based obesity prevention initiative | Used SNA to understand how community network structure influences obesity prevention capacity within 19 local government communities, prior to the rollout of the community-based obesity prevention intervention Healthy Together Victoria. | Network analysis |
| McGlashan et al 2016 | Quantifying a systems map: Network analysis of a childhood obesity causal loop diagram | Applied network analytic methods as a way of gaining quantitative insight into the structure of an obesity causal loop diagram to inform intervention design. | Network analysis |
| Meisel et al 2014 | Network analysis of bogotá’s ciclovía recreativa, a self-organized multisectorial community program to promote physical activity in a middle-income country | Conducted a SNA of the health and non-health related organisations that participated in Bogota’s Ciclovía Recreativa (Ciclovía). | Network analysis |
| Buck et al 2019 | Factors influencing sedentary behaviour: A system based analysis using Bayesian networks within DEDIPAC | Used Bayesian network analysis to investigate the clustering and interplay between factors that may be associated with SB across the lifecourse, in turn providing empirical validation of a previously developed sedentary behaviours (SOS)-framework, which was grounded in a systems-based approach. | System modelling |
| Anrooij et al 2020 | Improving environmental capacities for health promotion in support settings for people with intellectual disabilities: Inclusive design of the DIHASID Tool | Formative work to inform the development of a systems approach, involving the development of an environmental asset mapping tool in collaboration with people with intellectual disabilities to gain insight into existing assets for healthy nutrition and physical activity in support settings. | System framing |
| Apostolopoulos et al 2016 | The obesogenic environment of commercial trucking: A worksite environmental audit and implications for systems-based interventions | Provided a preliminary discussion on how complexity science can help design more efficacious interventions in the context of commercial trucking worksites and their influence on truck drivers’ opportunities for active living (and healthy eating). | System framing |
| Beets et al 2013 | Translating policies into practice: a framework to prevent childhood obesity in afterschool programs | Drew on complex systems theory to describe the development of a framework that identifies critical modifiable levers within afterschool programs that can be altered or strengthened to reach policy goals to combat childhood obesity. | System framing |
| Danks et al 2021 | Towards a common purpose: a theoretical model for a whole system approach to physical activity developed in South Tees | Developed a ‘Common Purpose Model’ to guide working practices and learning as part of the realist process evaluation of the Local Delivery Pilots commissioned by Sport England to tackle physical inactivity using a whole systems approach. The Model aims to provide a framework to support stakeholders with managing complexity when promoting the ‘You’ve Got This’ vision ‘active lives as a way of life’. | System framing |
| Economos and Tovar 2012 | Promoting health at the community level: thinking globally, acting locally | Described discussions of community-based methodologies (e.g. ANGELO (Analysis Grid for Environments Linked to Obesity) framework), best practice principles and a whole system intervention approach to obesity prevention. | System framing |
| Garcia et al 2017 | Development of a dynamic framework to explain population patterns of leisure-time physical activity through agent-based modeling | Created a conceptual model to inform and support the development of an ABM to explore interactions between psychological traits, built and social environments on LTPA. | System framing |
| Gortmaker et al 2011 | Obesity 4 Changing the future of obesity: science, policy, and action | Commentary that explored the role of systems science in providing a framework for organising the complex influences on obesity and identified priority actions including to improve built environments for PA. | System framing |
| Jess et al 2016 | Primary physical education: a complex learning journey for children and teachers | Developed a conceptual framework for primary physical education (PPE) based on ideas from complexity thinking. Explored how key principles including self-organisation, emergence, similarity, diversity, connectedness, nestedness, ambiguous bounding, recursive elaboration and edge of chaos, offered a lens for PPE as a complex system. | System framing |
| Johnston et al 2014 | Systems science and obesity policy: a novel framework for analyzing and rethinking population-level planning | Used the intervention-level framework (ILF) — described as a systems-based framework — to analyse obesity strategies and policy. | System framing |
| Jones et al 2014 | Chapter 3: Feasibility study of comprehensive school physical activity programs in Appalachian communities: The McDowell CHOICES Project | Evaluated the feasibility of a comprehensive school PA program (CSPAP) using a systems approach that considered the school, the system in which it operates, and its relationship with the environment. | System framing |
| Kohl et al 2012 | The pandemic of physical inactivity: global action for public health | Commentary about the need for a systems approach to increase PA worldwide, and a call to action for different ministries, countries, international bodies. | System framing |
| Lee et al 2017 | A systems approach to obesity | Discussed five key efforts to implement a systems approach for obesity prevention, with an example of how a systems approach had been used to convene a multidisciplinary team and conduct systems mapping and modelling for obesity in Baltimore. | System framing |
| Macmillan et al 2020 | Suburb-level changes for active transport to meet the SDGs: Causal theory and a New Zealand case study | Developed a complex dynamic causal theory for how equity-focused, participatory urban planning for walking and cycling contributes to specific United Nations’ Sustainable Development Goals, and used the Te Ara Mua Future Streets project as a case study to illustrate these pathways. | System framing |
| Mash 2010 | Chronic diseases, climate change and complexity: The hidden connections | Presented arguments about the hidden connections between the drivers behind chronic disease and climate change. Considered four key areas for disturbing the climate and chronic disease systems in South Africa to have healthier and more sustainable emergent properties: disturbing the network, the technology, the social structures and rules, and the meaning manifested in the system. | System framing |
| Nau et al 2019 | Toward whole-of-system action to promote physical activity: A cross-sectoral analysis of physical activity policy in Australia | Conducted a cross-sectoral analysis of PA-relevant policy in Australia. | System framing |
| Percival 2015 | Creating Healthy Places: a whole system approach to food and active living | Described the development of the ‘Creating Healthy Places’ workshop for local authorities to support a whole system approach for creating healthier and more sustainable communities. | System framing |
| Rigby et al 2020 | Challenges, opportunities and solutions for local physical activity stakeholders: an implementation case study from a cross-sectoral physical activity network in Northeast England | Conducted workshops with a cross-sectoral network of practice partners, policy makers, and researchers to identify the local-level challenges and opportunities for implementing PA policies and programmes. | System framing |
| Rütten et al 2019 | Co-producing active lifestyles as whole-system-approach: theory, intervention and knowledge-to-action implications | Outlined a theory-based system model for how to promote the co-production of active lifestyles as a whole system approach. Discussed four intervention models and used case studies from the German research network Capital4Health to illustrate how the models might work in practice. | System framing |
| Speake et al 2016 | Embedding physical activity in the heart of the NHS: The need for a whole-system approach | Explored what role the UK National Health Service (NHS) plays in a systems approach to population PA. Used the National Centre for Sport and Exercise Medicine (Sheffield) as a case study to discuss ways in which health systems can work collaboratively with other partners to promote active lifestyles. | System framing |
| Trowbridge et al 2013 | Public health and the green building industry partnership opportunities for childhood obesity prevention | Outlined how increased collaboration between public health and the green building industry can help increase consideration of health outcomes and drive positive change in built environment design and real estate investment. Outlined potential applications of using systems science to studying PA environments. | System framing |
| van Ommen et al 2018 | From diabetes care to diabetes cure-The integration of systems biology, eHealth, and behavioral change | Discussed how type 2 diabetes is a systems disease requiring a systems solution across the social, physiological and healthcare systems. | System framing |
| Zurcher et al 2018 | Food & Fitness: Lessons learned for funders | Reported on lessons learned for funders, from a 9-year W.K. Kellogg Foundation funded ‘Food & Fitness’ initiative to address emerging concerns about childhood obesity and health inequities from a system perspective. | System framing |
| Jancey et al 2019 | Exploring network structure and the role of key stakeholders to understand the obesity prevention system in an Australian metropolitan health service: study protocol | Presented a protocol for trialling a systems thinking approach to better understand the local obesity prevention system and identify gaps and opportunities for health promotion activities (including for PA) to strengthen obesity prevention efforts. Proposed using a systems inventory audit, organisational network survey and SNA. | Protocol development |
| Frerichs et al 2018 | Development of a systems science curriculum to engage rural African American teens in understanding and addressing childhood obesity prevention | Developed and tested a systems science curriculum to elicit rural African American youth perspectives on childhood obesity and enhance their understanding of and support for obesity prevention solutions. Conducted workshops with youth that engaged them in systems learning activities. | Methods development |
| Hoehner et al 2015 | Behavior-Over-Time Graphs: Assessing perceived trends in healthy eating and active living environments and behaviors across 49 communities | Conducted GMB sessions in the evaluation of Healthy Kids, Healthy Communities to promote systems thinking at the community level. As part of these sessions, participants created BOTGs to characterise stakeholder perceptions of changes in their community over time, relating to policies, environments, collaborations and social determinants for active living, healthy eating, and childhood obesity. This article focused on describing the methodological process for coding and analysing the BOTG trends generated over a large scale among diverse communities. | Methods development |
| Morris et al 2018 | Can big data solve a big problem? Reporting the obesity data landscape in line with the Foresight obesity system map | Mapped data sources against the Foresight obesity systems map domains and nodes to develop a framework of big data sources for use in whole systems obesity research. | Methods development |
| Sawyer et al 2021 | Developing the logic framework underpinning a whole-systems approach to childhood overweight and obesity prevention: Amsterdam Healthy Weight Approach | Formally documented the program theory of Amsterdam Healthy Weight Approach (AHWA), a long-term municipality-led program to improve children’s PA, diet, sleep through action in the home, neighbourhood, school and city that commenced in 2013. The logic framework is described as making explicit, the working principles of the whole systems approach underpinning AHWA. | Methods development |
| Wilkins et al 2020 | Evidence from big data in obesity research: international case studies | Presented three case studies to show how big data could be used to capture a broader range of variables in the obesity system, the specific benefits, limitations and challenges. One case study investigated the influence of the built environment on PA using spatial data on green spaces and exercise facilities alongside individual-level data on PA and swipe card entry to leisure centres. | Methods development |
| Brand 2017 | Whole-system approaches to improving the health and wellbeing of healthcare workers: A systematic review | Conducted a systematic review to identify ‘whole-system’ healthy workplace interventions in healthcare settings that incorporated the recommendations of the UK Department of Health regarding whole-system changes to improve healthcare staff health and wellbeing. | Literature synthesis |
| Essay et al 2021 | A scoping review of whole-of-community interventions on six modifiable cancer prevention risk factors in youth: A systems typology | Conducted a scoping review to synthesise the whole-of-community intervention literature on six modifiable risk factors in youth for cancer prevention, including PA, and to develop and apply a typology describing the fundamental functions needed for community system coordination. | Literature synthesis |
| Frerichs et al 2019 | A scoping review of simulation modeling in built environment and physical activity research: Current status, gaps, and future directions for improving translation | Used a scoping review framework to assess the use of simulation modelling to inform decision-making about built environment influences on PA. | Literature synthesis |
| Heine et al 2021 | Developing a complex understanding of physical activity in cardiometabolic disease from low-to-middle-income countries—A qualitative systematic review with meta-synthesis | Conducted a qualitative systematic review to obtain a comprehensive, systems-based overview of factors that affect PA in patients with cardiometabolic disease living in LMICs. A systems map of factors is presented in their findings. | Literature synthesis |
| Morshed et al 2019 | A systematic review of system dynamics and agent-based obesity models: Evaluating obesity as part of the global syndemic | Conducted a systematic review of SDM and ABM for obesity, to determine to what extent existing models provide insights into the shared drivers of the global syndemic of obesity, undernutrition and climate change. | Literature synthesis |
| Nianogo and Arah 2015 | Agent-based modeling of noncommunicable diseases: a systematic review | Conducted a systematic review of the use of ABMs to understand NCDs and their public health risk factors. | Literature synthesis |
| Panter et al 2017 | Physical activity and the environment: conceptual review and framework for intervention research | Conducted a literature synthesis of the perspectives and conceptual issues discussed or used in evaluative studies of interventions to change the physical environment. | Literature synthesis |
| Stankov et al 2020 | A systematic review of empirical and simulation studies evaluating the health impact of transportation interventions | Conducted a systematic review of systems-based simulation studies evaluating the health-related consequences of walking, cycling, aerial tram, bus and Bus Rapid Transit use. | Literature synthesis |
| Tracy et al 2018 | Agent-based modeling in public health: Current applications and future directions | Reviewed areas in public health where ABM has been adopted, their strengths and limitations, and future directions to enhance ABM for public health. | Literature synthesis |
| Xue et al 2018 | Applications of systems modelling in obesity research | Reviewed the applications of SDM and ABM in obesity research, examining how they were developed and used, and discussing related gaps. | Literature synthesis |
| Yang 2017 | Using agent-based modeling to study multiple risk factors and multiple health outcomes at multiple levels | Discussed the potential use of ABM for conducting ‘3M’ studies (studies that investigate how multiple risk factors influence multiple health outcomes at multiple levels). | Literature synthesis |

ABM=agent-based modelling; BMI=Body Mass Index; BOTG=behaviour-over-time graphs; CLD=causal loop diagram; FMS=Fundamental Movement Skill; GAPPA=The World Health Organization’s Global Action Plan on Physical Activity 2018-2030; GMB=group model building; LMIC=low-to-middle income countries; LTPA=leisure-time physical activity; NCD=noncommunicable disease; PA=physical activity; SB=sedentary behaviour; SDM=systems dynamic modelling.

#### Supplementary Table 2. Summary of included studies classified as ‘Prediction’.

| Author(s) and year | Title | Description of the application of a systems approach for PA | Methodological approach |
| --- | --- | --- | --- |
| PREDICTION |  |  |  |
| Macmillan and Woodcock 2017 | Understanding bicycling in cities using system dynamics modelling | Used participatory SDM to develop CLDs of cycling in 3 different contexts (Auckland, London, Nijmegen) to compare and contrast influences on cycling and draw out policy insights. | System mapping |
| Macmillan et al 2016 | Trends in local newspaper reporting of London cyclist fatalities 1992-2012: the role of the media in shaping the systems dynamics of cycling | As part of a wider effort to model the system dynamics of urban cycling, examined how media coverage of cyclist fatalities in London changed across a period of growth in cycling prevalence, and potential implications as a negative feedback loop on cycling participation. | System mapping |
| Giabbanelli et al 2012 | Modeling the influence of social networks and environment on energy balance and obesity | Developed a model for obesity that accounted for social and environmental influences on food and PA. | Network analysis |
| Abidin and Jamil 2016 | Simulation analysis on the consequences of behavioural change towards combating obesity: System dynamics approach | Simulated the effect of behavioural changes of eating and sedentary behaviour on weight, to create a weight behaviour model system that integrated information from PA, nutrition and body metabolism. | System modelling |
| Abidin et al 2014 | The role of physical activity to control obesity problem in Malaysia | Developed a SDM called SIMULObese, simulating the changes in PA in a Malaysian adult population to highlight its implications on body weight and prevalence of overweight and obesity. | System modelling |
| Abidin et al 2017 | A system dynamics optimization framework to achieve population desired of average weight target | Used a system dynamics approach of a stocks and flows diagram to quantitatively model the impact of changes to PA behaviours on weight and obesity. | System modelling |
| Almagor et al 2021 | How can an agent-based model explore the impact of interventions on children’s physical activity in an urban environment? | Explored the potential impact of interventions on PA among children by using an ABM simulating children’s daily activities in an urban environment. Three domains for interventions were explored: outdoor play, school physical education and active travel. | System modelling |
| Aziz et al 2018 | A high resolution agent-based model to support walk-bicycle infrastructure investment decisions: A case study with New York City | Built an ABM to assess the impact of changes in walk-bike infrastructures on walking and cycling. | System modelling |
| Aziza et al 2016 | SimNCD: An agent-based formalism for the study of noncommunicable diseases | Proposed a generic interaction-oriented ABM for NCDs, called SimNCD which models individuals living within a social network and daily engaging in activities from the physical environment. Also proposed a specific version of SimNCD for modelling childhood obesity, called SimNCDChO which models the complex relationships between children’s PA and the development of obesity. | System modelling |
| Badland et al 2013 | Using simple agent-based modeling to inform and enhance neighborhood walkability | Developed an open-source, simple agent-based tool that could be used to test scenarios for improving the walkability of neighbourhood catchments around actual or potential nodes of interest (e.g. schools, public transport stops). | System modelling |
| Bajracharya 2016 | Public transportation and private car: A system dynamics approach in understanding the mode choice | Developed a causal feedback loop model to study individual mode choice (public transport vs private car) in the context of Dubai, which was then translated into a SDM. | System modelling |
| Brittin et al 2021 | An agent-based simulation model for testing novel obesity interventions in school environment design | Developed an ABM to simulate the impact of using dynamic furniture in the school environment on PA in school children, and therefore childhood obesity. | System modelling |
| Cai and Liang 2021 | System dynamics modeling for a public-private partnership program to promote bicycle-metro integration based on evolutionary game | Developed a SDM for a public-private partnership program to promote bicycle-metro integration based on an evolutionary game model considering bike sharing companies, rail transit companies and long-distance travellers. | System modelling |
| Carrete et al 2017 | A socioecological view toward an understanding of how to prevent overweight in children | Developed a SDM to investigate the interrelationships between influences of socioenvironmental factors on PA and diet of children; and used it to analyse several social marketing scenarios on reducing the prevalence of overweight and obesity in children. | System modelling |
| Crielaard et al 2020 | Social norms and obesity prevalence: From cohort to system dynamics models | Constructed SDM based on an expert-informed CLD and data from six sociocultural groups to explore the effect of different scenarios on group level BMI. | System modelling |
| Ercan et al 2017 | Public transportation adoption requires a paradigm shift in urban development structure | Developed a SDM to model and test the critical system parameters affecting public transport usage in the United States and identify possible policy areas to improve public transport usage. | System modelling |
| Garcia et al 2018 | Exploring the emergence and evolution of population patterns of leisure-time physical activity through agent-based modelling | Developed an ABM to explore how interactions between psychological attributes and built and social environments may lead to the emergence and evolution of LTPA patterns among adults. | System modelling |
| Hekler et al 2013 | Exploring behavioral markers of long-term physical activity maintenance: A case study of system identification modeling within a behavioral intervention | Developed a SDM to explore whether particular intervention components (e.g. self monitoring, access to an exercise facility, behavioural initiation training) predicted differential patterns of behaviour among PA maintainers and non-maintainers. | System modelling |
| Honeycutt et al 2015 | Strategic planning for chronic disease prevention in rural America: Looking through a PRISM lens | Used the Prevention Impacts Simulation Model (PRISM) (an SDM) to project the reduction (compared to status quo) in deaths and costs from implementing a range of interventions (including for PA) to address chronic disease in the Mississippi Delta. | System modelling |
| Jin and Roger 2012 | An agent-based model of the influence of neighbourhood design on daily trip patterns | Developed an ABM to explore interactions between personal characteristics and preferences, and neo-traditional and fused grid designs, on outcomes such as automobile reliance, air pollution, traffic congestion, social interactions, and pedestrian benefits. | System modelling |
| Kuo et al 2016 | Framing the local context and estimating the health impact of CPPW obesity prevention strategies in Los Angeles County, 2010-2012 | Used the Prevention Impacts Simulation Model (PRISM) (an SDM) to forecast health impacts of obesity prevention efforts targeting PA promotion, healthy marketing and creation of healthy food environments. | System modelling |
| Lan et al 2014 | An investigation of factors affecting elementary school students’ BMI values based on the system dynamics modeling | Developed a SDM to investigate factors affecting school students’ BMI. | System modelling |
| Lee et al 2017 | Modeling the economic and health impact of increasing children’s physical activity in the United States | Developed a two-stage computational simulation model to quantify the economic and health impact of increasing PA. | System modelling |
| Lemoine et al 2016 | Using agent based modeling to assess the effect of increased Bus Rapid Transit system infrastructure on walking for transportation | Used an ABM to examine the effect of transport infrastructure on walking. | System modelling |
| Liu et al 2010 | Integrated simulation and optimization approach for studying urban transportation-environment systems in Beijing | Developed an SDM to simulate different scenarios combining policies on clean transport, bus priority, subway priority and car trip restriction, and effect on environmental and land use demand in Beijing. | System modelling |
| Macmillan et al 2014 | The societal costs and benefits of commuter bicycling: simulating the effects of specific policies using system dynamics modeling | Used SDM to compare the effects of policies to increase cycling in a car-dominated city and explored the role of participatory modelling to support transport planning. PA was one of the outcomes simulated. | System modelling |
| Masiolionyte et al 2020 | Estimating the impact of lifestyle changes on treatment outcomes for people with knee osteoarthritis through system dynamics simulation modelling | Developed a SDM to understand the potential impact of advanced lifestyle treatment (comprising classes delivered by musculoskeletal specialist with emphasis on changes in lifestyle such as joint protection) on osteoarthritis outcomes. | System modelling |
| McClure et al 2015 | Simulating the dynamic effect of land use and transport policies on the health of populations | Developed a SDM to represent relationships among land use, transport, economic development, and population health as a way of demonstrating the implications of replacing the focused policy question about road fatalities, with the more holistic question about the features of a land use-transportation system that optimise the health and wellbeing of the population. | System modelling |
| McDonnell and Zellner 2011 | Exploring the effectiveness of bus rapid transit a prototype agent-based model of commuting behavior | Developed a prototype ABM to test the effects of different Bus Rapid Transit policy changes on modal share. | System modelling |
| Mehrjerdi et al 2020 | Dynamic analysis of health-related factors with its impacts on economic growth | Developed an SDM to investigate the impacts of health-related factors on economic growth of Iran. Scenarios tested included those associated with PA. | System modelling |
| Millard 2012 | The obesity pandemic: Implementing the evidence for children in Scottish families | Applied an environmental health systems modelling approach – the Drivers, Pressures, State, Exposure, Effects, Actions (DPSEEA) model – to child obesity in Scotland. | System modelling |
| Montes et al 2012 | Do health benefits outweigh the costs of mass recreational programs? An economic analysis of four Ciclovía programs | Conducted an analysis of the cost-benefit ratios of PA of the Ciclovía programs of Bogotá and Medellín in Colombia, Guadalajara in México, and San Francisco in the USA. | System modelling |
| Okushima and Akiyama 2011 | Multi-agent transport simulation model for eco-commuting promotion planning | Developed a multi-agent simulation system consisting of three interactive models – an agent travel behaviour model, traffic environment model, and agent eco-consciousness model to enable analysis of the influence of interventions to promote eco-commuting, on modal shift. | System modelling |
| Okushima 2015 | Simulating social influences on sustainable mobility shifts for heterogeneous agents | Developed a multi-agent mobility shift model to examine the dynamics of commuting mode choice and purchase of clean energy vehicles. | System modelling |
| Orr et al 2016 | Neighbourhood food, physical activity, and educational environments and black/white disparities in obesity: a complex systems simulation analysis | Used an ABM to simulate the effects of PA infrastructure (and other interventions) on reducing black/white disparities in BMI. | System modelling |
| Papageorgiou and Demetriou 2019 | Investigating learning and diffusion strategies for sustainable mobility | Developed a SDM to test learning strategies that promote walking. A case study was used to contextualise and demonstrate the active mobility issue to develop practical scenarios for promoting a walking mindset. | System modelling |
| Powell et al 2017 | Systems thinking and simulation modeling to inform childhood obesity policy and practice | Updated a SDM originally launched in 2008 that simulated the impact of policy interventions on the prevalence of childhood obesity in Georgia through to 2034. | System modelling |
| Powell-Wiley et al 2017 | Simulating the impact of crime on African American women's physical activity and obesity | Developed an ABM to quantify the impact of crime on PA location accessibility, LTPA and obesity among African American women under different circumstances. | System modelling |
| Salvo et al 2021 | Physical activity promotion and the United Nations Sustainable Development Goals: Building synergies to maximize impact | Examined the contribution of PA promotion strategies toward achieving the SDGs through a conceptual linkage exercise, a scoping review, and an ABM. | System modelling |
| Shoham et al 2012 | An actor-based model of social network influence on adolescent body size, screen time, and playing sports | Used a stochastic actor-based model framework to model network dynamics (friendship selection) and social influence among adolescents, and their effect on playing active sports and screen time. | System modelling |
| Soler et al 2016 | Community-based interventions to decrease obesity and tobacco exposure and reduce health care costs: Outcome estimates from Communities Putting Prevention to Work for 2010–2020 | Used a mixed-methods approach to estimate population reach and simulate the effects of completed interventions from the Centers for Disease Control and Prevention 'Communities Putting Prevention to Work (CPPW)' program. | System modelling |
| Tonini et al 2021 | The dynamics of individual behaviour of mode choice: The impacts of selected Brazilian urban mobility Policy' instruments | Developed a SDM to evaluate the impact of changes in selected urban conditions on individual behaviours in terms of transport mode choice, through the implementation of an urban mobility policy. | System modelling |
| Wen and Bai 2017 | System dynamics modeling and policy simulation for urban traffic: a case study in Beijing | Conducted a SDM for simulating the impact of different strategies on urban traffic's energy consumption and carbon emissions in Beijing. | System modelling |
| Yang and Diez-Rouz 2013 | Using an agent-based model to simulate children’s active travel to school | Used an ABM to simulate children's school travel behaviour within a hypothetical city, to explore plausible implications of policies targeting long distance to school and traffic safety. | System modelling |
| Yang et al 2011 | A spatial agent-based model for the simulation of adults' daily walking within a city | Developed a spatial ABM to simulate walking behaviours within a city and examine the contribution of land use and safety to socioeconomic differences in walking. | System modelling |
| Yang et al 2012 | Exploring walking differences by socioeconomic status using a spatial agent-based model | Used an exploratory ABM of adults' walking behaviour within a city to examine the possible impact of interventions on socioeconomic differences in walking. | System modelling |
| Yang et al 2014 | Examining the impact of the walking school bus with an agent-based model | Used an ABM to examine the impact of a walking school bus on children's active travel to school. | System modelling |
| Yang et al 2015 | Modeling spatial segregation and travel cost influences on utilitarian walking: Towards policy intervention | Developed an ABM of utilitarian walking to explore spatial and socioeconomic factors affecting this outcome and how travel costs and educational interventions can alter the prevalence and income differentials in walking. | System modelling |
| Yang et al 2019 | Is the decline of active travel to school unavoidable by-products of economic growth and urbanization in developing countries? | Developed a SDM to study active travel to school in China and test possible effects of changing one or more factors (re economic development, urban sprawl, urban design, crime), and considering their dynamic interrelationship. | System modelling |
| Yarnoff et al 2019 | Estimating the relative impact of clinical and preventive community-based interventions: An example based on the Community Transformation Grant program | Used the Prevention Impacts Simulation Model (PRISM) to simulate the potential 10- and 25- year impacts (premature deaths averted, health care cost savings, risk factor management cost savings) of large scale clinical and community interventions as they were implemented as part of the Community Transformation Grant (CTG) program. | System modelling |
| Yin 2013 | Assessing walkability in the City of Buffalo: An application of agent-based simulation | Used an ABM approach to explore the dynamics of the built environment and people's decision making processes concerning walking, in the context of Buffalo city. | System modelling |
| Zellner et al 2016 | Overcoming the last-mile problem with transportation and land-use improvements: an agent-based approach | Developed an ABM representing commuters and their preferences for different aspects of transport disutility (cost, time, safety), to explore how transport improvements and physical improvements enhancing active transport commute might overcome the last mile problem. | System modelling |
| Zhang et al 2015 | Network interventions on physical activity in an afterschool program: an agent-based social network study | Used SNA and ABM to test whether implementing a network intervention (i.e. using social network data) would increase children's PA, using a real world social network of children in low SES neighbourhoods. | System modelling |
| Zou et al 2016 | An agent-based choice model for travel mode and departure time and its case study in Beijing | Developed an ABM for travellers' choice of modes and departure time, to evaluate congestion charge policies with various demand scenarios. | System modelling |
| Potter and Ulijaszek 2013 | Predicting adult obesity from measures in earlier life | Constructed a two-way multifactor risk assessment framework for predicting adult obesity during childhood using the Foresight systems map and testing against longitudinal data. | Generic methods |
| Frerichs et al 2020 | Novel participatory methods for co-building an agent-based model of physical activity with youth | Co-created an ABM about PA with adolescent youth. | Methods development |
| Hennessy et al 2016 | Designing an agent-based model for childhood obesity interventions: A case study of ChildObesity180 | Developed an ABM to evaluate and refine implementation of behaviour change interventions to increase PA, healthy eating and reduce childhood obesity. | Methods development |
| Torrens et al 2012 | An extensible simulation environment and movement metrics for testing walking behavior in agent-based models | Examined popularly-used methods to drive movement in ABMs, by introducing a methodology that could flexibly handle many representations of movement at different scales, and to introduce a suite of tools to benchmark agent movement between models and against real-world trajectory data. | Methods development |

ABM=agent-based modelling; BMI=Body Mass Index; CLD=causal loop diagram; LTPA=leisure-time physical activity; PA=physical activity; SDM=systems dynamic modelling; SES=socioeconomic status; SNA=social network analysis

#### Supplementary Table 3. Summary of included studies classified as ‘Intervention development’.

| Author(s) and year | Title | Description of the application of a systems approach for PA | Methodological approach |
| --- | --- | --- | --- |
| INTERVENTION DEVELOPMENT |  |  |  |
| Brennan et al 2015 | Systems thinking in 49 communities related to healthy eating, active living, and childhood obesity | Presents evaluation methods and findings from 49 Healthy Kids, Healthy Communities sites funded to implement policy, system and environmental changes from 2008 to 2014. Evaluation involved GMB sessions between 2010 and 2013, and a total 50 CLDs for 49 communities. Analysis focused on the most prominent variables in CLDs across communities; major feedback structures; and implications from the synthesised CLD that could be translated to policy makers, practitioners, evaluators, funders, and other community representatives. | System mapping |
| Moreland 2015 | Improving park space access for the Healthy Kids, Healthy Communities partnership in Denver, Colorado | A GMB workshop was held with key members of the Denver Healthy Kids, Healthy Communities (HKHC) coalition to create BOTGs and a CLD, identifying factors that affect or are affected by policy, system and environmental changes that influence active living, healthy eating or childhood obesity. | System mapping |
| Amed et al 2016 | Wayfinding the Live 5-2-1-0 Initiative—At the intersection between systems thinking and community-based Childhood Obesity Prevention | Describes the protocol for implementing and evaluating the knowledge exchange model supporting the implementation of ‘Live 5-2-1-0’, a multisector, multicomponent childhood obesity prevention initiative informed by systems thinking and participatory research. The aim was to understand how to facilitate and sustain systems/community-level change. | System framing |
| Macmillan et al 2018 | Controlled before-after intervention study of suburb-wide street changes to increase walking and cycling: Te Ara Mua-Future Streets study design | Used qualitative SDM to develop a causal theory for the relationships between active travel, and walking and cycling infrastructure. Triangulated best evidence to develop interventions that were contextually and culturally appropriate. | System framing |
| Newman et al 2016 | Applying Health in All Policies to obesity in South Australia | Detailed how the Government of South Australia used the Health in All Policies (HiAP) approach in the SA HiAP Healthy Weight Project, with a focus on nutrition and PA. | System framing |
| Signal et al 2013 | Tackling 'wicked' health promotion problems: A New Zealand case study | Conducted multi-phase research using literature review, focus groups, stakeholder workshops and key informant interviews to identify possible policy interventions to enhance food security and PA among Maori, Pacific and low-income people in New Zealand. | System framing |
| Allender et al 2016 | Whole of Systems Trial of Prevention Strategies for childhood obesity: WHO STOPS Childhood Obesity | Presented a protocol for a stepped wedge cluster randomized trial in ten communities in the Great South Coast Region of Victoria, Australia to test whether it was possible to: (1) strengthen community action for childhood obesity prevention, and (2) measure the impact of increased action on risk factors for childhood obesity. The intervention would involve a facilitated community engagement process to create an agreed systems map of childhood causes of obesity; identify intervention opportunities; and convert these into community-built, systems-oriented action plans. | Protocol development |
| Maitland et al 2019 | Campbelltown - Changing our Future: study protocol for a whole of system approach to childhood obesity in South Western Sydney | Presented a protocol for the Campbelltown Changing our Future study which proposes to translate a whole of system approach previously trialled in rural Victoria and the Australian Capital Territory, to the Campbelltown local government area to address childhood obesity. | Protocol development |
| Vamos et al 2016 | Community-based pilot intervention to tackle childhood obesity: a whole-system approach | Described the methods and practical steps in the development of 'Go-Golborne', a pilot intervention using a multistrategy approach, to prevent childhood obesity in the Royal Borough of Kensington and Chelsea. | Protocol development |
| Waterlander et al 2020 | A system dynamics and participatory action research approach to promote healthy living and a healthy weight among 10-14-Year-old adolescents in Amsterdam: The LIKE Programme | Described the design and intended aims of the Lifestyle Innovations based on youth's Knowledge and Experience (LIKE) program – a public health intervention to tackle childhood overweight and obesity – using a systems dynamics and participatory action approach. | Protocol development |
| Watts et al 2020 | The long-term health and wellbeing impacts of Healthy New Towns (HNTs): protocol for a baseline and feasibility study of HNT demonstrator sites in England | Described a protocol for a baseline and feasibility study of the Healthy New Town demonstrator sites in England, including the use of systems thinking methods such as systems mapping. | Protocol development |

CLD=causal loop diagram; GMB=group model building; PA=physical activity.

#### Supplementary Table 4. Summary of included studies classified as ‘Process evaluation’.

| Author(s) and year | Title | Description of the application of a systems approach for PA | Methodological approach |
| --- | --- | --- | --- |
| PROCESS EVALUATION |  |  |  |
| Nobles et al 2019 | Understanding how local authorities in England address obesity: A wider determinants of health perspective | Aimed to understand what actions were being taken by local government organisations to address obesity, and determine how they countered the perceived causes of obesity when mapped against the Wider Determinants of Health model. | System mapping |
| Allender et al 2019 | Translating systems thinking into practice for community action on childhood obesity | Reported on the first 18 months of two communities' efforts using methods inspired by community-based participatory systems dynamics for the development, implementation, and evaluation of whole of community efforts to improve children's health. Applied the Foster-Fishman's theoretical framework for characterising systems change to describe the initiatives and make sense of the initial effort. | System framing |
| Bartelink 2019 | Process evaluation of the Healthy Primary School of the Future: the key learning points | Explored the processes through which the Healthy Primary School of the Future (HPSF) initiative and the school context adapted to and influenced one another, using a programme theory which was based on a contextual action-oriented research approach and complex systems thinking. | System framing |
| Knai et al 2018 | The Public Health Responsibility Deal: Using a systems-level analysis to understand the lack of impact on alcohol, food, physical activity, and workplace health sub-systems | Uses a systems approach to analyse the main findings and implications of an evaluation of the Public Health Responsibility Deal (RD) (a public-private partnership in England), including to explore how the RD interacted with the PA system. | System framing |
| Matheson et al 2020 | Strengthening prevention in communities through systems change: lessons from the evaluation of Healthy Families NZ | Presented the findings from the first 3 years of the evaluation of Healthy Families NZ (a systems-change intervention to prevent chronic disease in 10 communities). The initiative targeted PA among other things. The evaluation covered the ways in which the prevention system had changed in terms of prevention infrastructure, and prevention attitudes and paradigms. | System framing |
| O'Tuama 2015 | Ripples through the city: Understanding the processes set in motion through embedding a public bike sharing scheme in a city | Aimed to elucidate the nature of the processes or 'ripple effects' associated with embedding a public bike sharing scheme into the physical, social and institutional fabric of a city, using the ‘dublinbikes’ scheme as a case study. | System framing |
| Potts et al 2021 | Working with local people as part of a whole-systems approach to physical activity: reflections from local delivery pilots | Provided reflections from researchers embedded within two local delivery pilots commissioned by Sport England, around efforts to work with local people as part of a whole systems approach to enable active lives. | System framing |
| Sautkina et al 2014 | Lost in translation? Theory, policy and practice in systems-based environmental approaches to obesity prevention in the Healthy Towns programme in England | Explored how system-wide approaches to obesity prevention were theorised and translated into practice in the Healthy Towns program. | System framing |
| Hall et al 2021 | A whole system approach to increasing children's physical activity in a multi-ethnic UK city: a process evaluation protocol | Described the protocol for a process evaluation of the JU:MP programme, a whole systems approach to increasing PA in children and young people aged 5-14 years in North Bradford, UK. Proposed a mixed methods data collection approach that would include semi-structured interview, observation, documentary analysis, surveys, and participatory evaluation methods including reflections and ripple effect mapping. | Protocol development |
| Serpas et al 2013 | San Diego Healthy Weight Collaborative: a systems approach to address childhood obesity | Described a process of aligning multiple partners in primary care, public health, university research, schools and community organisations, to address childhood obesity in a Latino underserved community (San Diego Healthy Weight Collaborative). The paper analysed the implementation process, relevant outcomes, sustainability efforts and lessons learned from each of the key strategies. | Generic methods |
| Whelan et al 2019 | A rural community moves closer to sustainable obesity prevention - an exploration of community readiness pre and post a community-based participatory intervention | Evaluated community readiness to change, to inform and evaluate the impact of a whole of community, systems level obesity prevention initiative (YCHANGe) in a rural community in Victoria. | Generic methods |

PA=physical activity.

#### Supplementary Table 5. Summary of included studies classified as ‘Impact evaluation’.

| Author(s) and year | Title | Description of the application of a systems approach for PA | Methodological approach |
| --- | --- | --- | --- |
| IMPACT EVALUATION |  |  |  |
| Allender et al 2021 | Four-year behavioral, health-related quality of life, and BMI outcomes from a cluster randomized Whole of Systems Trial of Prevention Strategies for Childhood Obesity | Evaluated the effectiveness of the Whole of Systems Trial of Prevention Strategies for Childhood Obesity (WHO STOPS Childhood Obesity) for PA and other outcomes (behavioural, health-related quality of life, and BMI). | Generic methods |
| Bartelink et al 2019 | Can the Healthy Primary School of the Future offer perspective in the ongoing obesity epidemic in young children? A Dutch quasi-experimental study | Assessed the effect of the Healthy Primary School of the Future initiative, described as using a ‘contextual systems approach’, on children's BMI after 1 and 2 years’ follow-up. | Generic Methods |
| Folta et al 2013 | Changes in diet and physical activity resulting from the Shape Up Somerville community intervention | Evaluated the behavioural changes (including in PA) in children resulting from Shape Up Somerville which was described as a community-based, participatory obesity prevention intervention using a multilevel, systems-based approach. | Generic methods |
| Jacobs et al 2021 | The impact of a community-based intervention on weight, weight-related behaviours and health-related quality of life in primary school children in Victoria, Australia, according to socio-economic position | Conducted secondary analysis on data collected from primary school children in 10 communities involved in the Whole of Systems Trial of Prevention Strategies for Childhood Obesity (WHO STOPS) cluster randomised trial in Victoria, Australia. Outcomes included self-reported PA. | Generic methods |
| Lane et al 2021 | Health promoting sports club in Practice: A controlled evaluation of the GAA Healthy Club Project | Assessed and described the health promotion impact and experience of the Gaelic Athletic Association Healthy Club Project, described as a health promotion activity guided by systems thinking in a sport setting. | Generic methods |
| Malakellis et al 2017 | School-based systems change for obesity prevention in adolescents: outcomes of the Australian Capital Territory 'It's Your Move!' | Conducted an evaluation of the Australian Capital Territory 'It's Your Move!', a 3-year school-based systems change intervention to prevent obesity in adolescents. | Generic methods |
| Schwarte et al 2010 | The Central California Regional Obesity Prevention Program: changing nutrition and physical activity environments in California's heartland | Evaluated the Central California Regional Obesity Prevention Program which aimed to promote safe places for PA, and support youth engagement in efforts to change environments for obesity prevention. | Generic methods |
| Bagnall et al 2019 | Whole systems approaches to obesity and other complex public health challenges: a systematic review | Conducted a systematic review of whole systems approaches targeting obesity and other complex public health and societal issues, such as healthy lifestyles for prevention of NCDs. Analysis included identifying the elements of a whole systems approach that were effective (or not) in obesity and other areas of public health. | Literature synthesis |

BMI=Body Mass Index; NCD=noncommunicable disease; PA=physical activity.

#### Supplementary Table 6. Summary of included studied classified as ‘Unclear systems approaches’.

| Author(s) and year | Title | Description of the application of a systems approach for PA |
| --- | --- | --- |
| Brannan et al 2019 | Moving healthcare professionals - a whole system approach to embed physical activity in clinical practice | Provides an overview of the Moving Healthcare Professionals program (MHPP) - a whole system educational approach to embed prevention and PA into clinical practice. |
| Simione et al 2021 | Effects of the First 1000 Days Program, a systems-change intervention, on obesity risk factors during pregnancy | Examined changes from the first to third trimester for women participating in the First 1000 Days Program, described as a systems-oriented program starting in early pregnancy and lasting through the first 24 months of infancy that was focused on preventing obesity and related risk factors among low income, mother-infant pairs. |
| Smith et al 2019 | Active residents in care homes: A holistic approach to promoting and encouraging meaningful activity for residents living in care homes: Innovative Practice | Reported on the Active Residents in Care Homes intervention (described as a whole systems approach to promote meaningful activity among care home residents), its evaluation methods and preliminary findings. |

PA=physical activity.
